# Dynamic changes in gene expression *in vivo *predict prognosis of tamoxifen-treated patients with breast cancer

**DOI:** 10.1186/bcr2593

**Published:** 2010-06-22

**Authors:** Karen J Taylor, Andrew H Sims, Liang Liang, Dana Faratian, Morwenna Muir, Graeme Walker, Barbara Kuske, J Michael Dixon, David A Cameron, David J Harrison, Simon P Langdon

**Affiliations:** 1 CRUK Cancer Research Centre and Academic Breast Unit, Institute of Genetics and Molecular Medicine, University of Edinburgh, Crewe Road South, Edinburgh, EH4 2XU, UK; 2 Applied Bioinformatics of Cancer Group, Institute of Genetics and Molecular Medicine, University of Edinburgh, Crewe Road South, Edinburgh, EH4 2XU, UK; 3 Breakthrough Breast Cancer Research Unit and Division of Pathology, Institute of Genetics and Molecular Medicine, University of Edinburgh, Crewe Road South, Edinburgh, EH4 2XU, UK; 4 NCRN Coordinating Centre, University of Leeds, MacMillan Wing, Fairbairn House, 71-75 Clarendon Road, Leeds, LS2 9PH, UK

## Abstract

**Introduction:**

Tamoxifen is the most widely prescribed anti-estrogen treatment for patients with estrogen receptor (ER)-positive breast cancer. However, there is still a need for biomarkers that reliably predict endocrine sensitivity in breast cancers and these may well be expressed in a dynamic manner.

**Methods:**

In this study we assessed gene expression changes at multiple time points (days 1, 2, 4, 7, 14) after tamoxifen treatment in the ER-positive ZR-75-1 xenograft model that displays significant changes in apoptosis, proliferation and angiogenesis within 2 days of therapy.

**Results:**

Hierarchical clustering identified six time-related gene expression patterns, which separated into three groups: two with early/transient responses, two with continuous/late responses and two with variable response patterns. The early/transient response represented reductions in many genes that are involved in cell cycle and proliferation (e.g. BUB1B, CCNA2, CDKN3, MKI67, UBE2C), whereas the continuous/late changed genes represented the more classical estrogen response genes (e.g. TFF1, TFF3, IGFBP5). Genes and the proteins they encode were confirmed to have similar temporal patterns of expression *in vitro *and *in vivo *and correlated with reduction in tumour volume in primary breast cancer. The profiles of genes that were most differentially expressed on days 2, 4 and 7 following treatment were able to predict prognosis, whereas those most changed on days 1 and 14 were not, in four tamoxifen treated datasets representing a total of 404 patients.

**Conclusions:**

Both early/transient/proliferation response genes and continuous/late/estrogen-response genes are able to predict prognosis of primary breast tumours in a dynamic manner. Temporal expression of therapy-response genes is clearly an important factor in characterising the response to endocrine therapy in breast tumours which has significant implications for the timing of biopsies in neoadjuvant biomarker studies.

## Introduction

The majority of human breast cancers express estrogen receptor alpha (ERα) and are estrogen responsive [1]. Tamoxifen is still the most widely prescribed anti-estrogen for patients with ER-positive breast cancer and has improved survival in women initially receiving this drug as adjuvant therapy [2]. However, although the majority of women respond to this agent, not all patients benefit and there is a need to identify with greater precision which tumors are sensitive and responding to this therapy. Dynamic changes in specific marker genes in biopsy material at early treatment points could be informative and might indicate whether a tumor is likely to regress or progress.

Although many *in vitro *studies have explored estrogen- and tamoxifen-regulated changes on gene expression [3-7], we are unaware of any xenograft studies that have investigated the temporal regulation of expression changes produced by tamoxifen in an ER-positive model *in vivo*. Previous attempts to characterize the gene expression response to tamoxifen in breast tumors *in vivo *have been limited to single time points [8,9]. A recent time course experiment demonstrated dynamic gene expression changes in response to estradiol (E_2_) in ZR-75-1 cell lines *in vitro *[10]. Xenograft models allow assessment of dynamic changes in tissue gene expression at multiple time points from tissue, which is not feasible in the clinical setting. Furthermore, an *in vivo *model allows the effect of stromal elements and matrix elements to contribute to expression, which cannot be easily reproduced *in vitro*.

A number of studies have investigated whether differences in gene expression in primary tumors (prior to treatment) are associated with or can predict the response to tamoxifen [11-13]. Vendrell and colleagues recently described a candidate molecular signature associated with tamoxifen failure in primary breast cancer by examining gene expression in tumors following tamoxifen treatment [14]. An alternative to measuring gene expression differences in the primary static situation is to compare tumor biopsies matched before and after treatment in so-called neoadjuvant 'window of opportunity studies' [15]; these are likely to generate interesting results in the near future.

We have previously used the ER-positive ZR-75-1 breast cancer xenograft model to demonstrate that tamoxifen causes significant changes in apoptosis, proliferation and angiogenesis within two days of initiating therapy, which both antedated any evidence of growth response and persisted for up to 14 days [3,16]. Here we present the first study to look at the dynamic changes in gene expression using multiple time points following treatment with tamoxifen *in vivo *in order to better understand the temporal response to therapy.

## Materials and methods

### Cell culture

The ZR-75-1, MCF-7 and MDA-MB-231 breast cancer cell lines were obtained from the American Type Culture Collection. Cells were maintained in DMEM (Life Technologies, Paisley, Scotland) containing 10% heat-inactivated FCS, penicillin (100 units/mL), and streptomycin (100 μg/mL). Cells were maintained routinely at 37°C in a humidified atmosphere of 5% carbon dioxide in air. Forty-eight hours before treatment, medium was changed to phenol-red-free DMEM containing 5% double charcoal stripped FCS, glutamine (2 mM), penicillin (100 U/ml) and streptomycin (100 μg/ml). For temporal analysis of gene expression, ZR-75-1, MCF-7 and MDA-MB-231 were incubated with 0.1 nM 17 β-E_2 _and/or tamoxifen (1 μM) (Sigma-Aldrich Chemical Co St. Louis, MO, USA) or in serum-free medium alone for 0, 6 and 24 hours.

### Xenograft experiment

All mouse experiments were performed in accordance with Home Office guidelines. For the xenograft studies, the ZR-75-1 cell line was first implanted into female nu/nu mice. Animals received a subcutaneous slow-release E_2 _pellet (0.72 mg released over 60 days, Innovative Research of America, Sarasota, FL, USA) on the day of tumor implant. The tumor was then maintained subcutaneously in the flanks of recipient animals by passaging 1 mm^3 ^fragments as required, approximately every eight weeks. For microarray experiments, ZR-75-1 fragments were implanted subcutaneously into animals and allowed to grow to a mean size of 0.25 cm^3^. All animals received E_2_. On day 0, animals were randomly allocated to tamoxifen (2.5 mg released over 60 days, Innovative Research of America, Sarasota, FL, USA) or E_2_-only control groups. There were 20 mice, with tumors in each flank, in both the control and treatment groups of this experiment. Tumor volumes were measured using vernier callipers. Bidimensional tumor diameters were recorded and volumes calculated as vol = πDd^2^/6, where D is the larger of the two diameters. Four mice from each group were sacrificed at each time point.

### RNA extraction

Tumor xenografts treated with E_2_-only or E_2 _and tamoxifen were obtained from animals sacrificed on days 0, 1, 2, 4, 7 and 14. These were homogenized in lysis buffer and total RNA was extracted using the Qiagen RNeasy^® ^kit (Qiagen, Valencia, CA, USA), according to the manufacturer's instructions. The concentration and purity of RNA were determined by measuring spectrophotometric absorption at 260 to 280 nm. To verify the integrity of the total RNA, the samples were electrophoresed on a 1% agarose gel in RNA loading buffer. A pool of total RNA from xenografts collected on days 0, 1, 2 and 4, treated with E_2 _only, was used as the reference population for all cDNA microarray hybridisations. This provided an internal standard when compared with each experimental sample.

### Probe preparation, labelling, hybridisation and scanning of microarrays

Total RNA (100 ug), spiked with a bacterial-RNA mixture for control, was used to prepare direct Cy3- and Cy5-labelled first-strand cDNA probes using a single-base anchored oligo dT17 primer (Sigma, St. Louis, MO, USA) and Superscript II reverse transcriptase (Invitrogen, Carlsbad, CA, USA). Unincorporated nucleotides were removed using QIAquick PCR purification kit (QIAGEN, Valencia, CA, USA) and Cy3- and Cy5-labelled probes were coprecipitated with 16 μg human Cot 1 DNA (Invitrogen, Carlsbad, CA, USA) and 8 μg polyA (Sigma, St. Louis, MO, USA). The pellets were resuspended in 8 μl of H_2_O and 40 μl of hybridization buffer (5 × Saline-Sodium Citrate (SSC), 6 × Denhardt's solution, 60 mM Tris HCl pH 7.6, 0.12% sarkosyl, 48% formamide) boiled for five minutes and cooled at room temperature for 10 minutes. The mix was overlaid with a coverslip and hybridized at 47°C for 12 to 24 hours in a humidified atmosphere to Sanger Hver 1.3.1 cDNA microarrays (Sanger, Cambridge, UK) as part of the CRUK/LICR Microarray Consortium, contain 9,930 sequence-validated cDNA clones representing approximately 6,000 unique sequences. Microarrays were washed sequentially with 2 × SSC, 0.1 × SSC/0.1% SDS, and 0.1 × SSC and were air-dried by briefly spinning in a centrifuge to remove excess liquid. Fluorescent images of hybridized microarrays were captured using a ScanArray Express 3.0 scanner and ScanArray software (both from Perkin Elmer Waltham, MA, USA).

### Analysis of microarray data

Comparisons were made between pooled E_2 _only-treated controls and E_2 _plus tamoxifen-treated samples across the following time points - days 1, 2, 4, 7 and 14 - and included reciprocal dye labelling to exclude gene-specific dye bias. Expression ratios (Cy5/Cy3) were calculated following background correction using the R programming language [17] and the BioConductor [18] package limma [19] to account for dye bias. Intensity dependant (Loess) and quantile normalization were also performed. Fold changes were calculated as the relative mean difference between treated and untreated dye-swap replicates. Normalized data and raw gene expression files are publicly available from in NCBI's Gene Expression Omnibus (GEO) [20] and are accessible through GEO Series accession number GSE22386. Clustering was performed using the Cluster and TreeView [21] programs. Kaplan Meier analysis was performed using SPSS version 14 (an IBM Company, Chicago, IL, USA). Estrogen-response elements (EREs) were identified using the Dragon program [22]. Genes with the greatest prognostic power were identified using supervized principle components analysis [23] using version 3.5 of BRB ArrayTools [24] as previously described [12]. The Database for Annotation, Visualization and Integrated Discovery (DAVID) [25] was used to identify KEGG (Kyoto Encyclopedia of Genes and Genomes) pathways and Gene Ontology terms that were significantly over-represented in gene lists above the level expected by chance.

### Validation of targets by quantitative RT-PCR

The expression of putative tamoxifen-regulated genes in the ER-positive cell lines ZR-75-1 and MCF7 and the ER-negative cell line MDA-MB-231 was performed by quantitative RT-PCR. Cells were maintained as outlined above. A specific set of primers was designed for each target [see Additional File 1]. Total RNA was extracted from log-phase cells using TRI reagent (Sigma, Poole, UK) following the manufacturer's instructions and treated with DNAse I (Roche, Indianapolis, IN, USA). RNA was analyzed by real-time RT-PCR using Rotorgene (Corbett Research, San Francisco, CA, USA) and the QuantiTect SYBR Green system (QIAGEN, Valencia, CA, USA) according to the manufacturers' instructions. Thermal cycling conditions were as follows: RT at 50°C for 30 minutes; PCR: polymerase activation 95°C for 15 minutes, followed by 40 cycles of denaturation at 94°C for 15 seconds, annealing at 57°C for 30 seconds and extension at 72°C for 45 seconds. After a final extension at 72°C for 5 minutes, the melt profile was obtained across a 65°C to 99°C ramp, with 5 second ramps of 1°C. All reactions were performed in triplicate for standard curve samples and in quadruplicate for experimental and negative (no template) samples. Analysis and quantification was performed using Rotorgene v6 software. Relative quantification was calculated by extrapolation of the standard curve and calculation of ratio levels compared with β-Actin.

### Immunohistochemistry

All experiments involving human tissues were conducted with the permission of the local medical ethics advisory board. A series of women over the age of 70 years with large operable or locally advanced primary breast cancer without metastatic disease presenting to the Edinburgh Breast Unit between October 1991 and October 1993 have previously been described [26]. All had tumors greater than 2 cm in maximum diameter, confirmed as ER-positive invasive breast cancer. All patients received 20 mg tamoxifen daily for three months. Tumor size was monitored by ultrasound measurements, and clinical response defined as the percentage volume reduction between the initial and final tumor volumes at three months.

Formalin fixed paraffin-embedded blocks from the initial wedge biopsy and at definitive loco-regional surgery three months later were available for 28 of these patients and 3 μm tissue sections were cut. Formalin-fixed paraffin embedded (FFPE) blocks were also available from the original parallel xenograft study, which analyzed proliferation and apoptosis changes after tamoxifen treatment in the ZR-75-1 xenograft [3]. Sections were deparaffinized and rehydrated by standard methods and endogenous peroxidase activity blocked by incubation in 3% H_2_O_2 _for 30 minutes. Sections were immersed in citrate buffer (0.005 M, pH 6.0) and microwaved three times for five minutes and then allowed to stand for 20 minutes. Slides were washed in 0.05 M Tris/NaCl buffer (pH 7.6) and then incubated in 20% FCS for 10 minutes prior to the addition of the primary antibodies at room temperature for 90 minutes in a humidified container. Optimal conditions for antigen retrieval, and primary antibody dilutions were previously determined for each antibody, as follows: trefoil factor (TFF) 3 (1/5, Calbiochem, Nottingham, UK), PDZK1 (1/10, Abnova, Taipei City Taiwan), insulin growth factor receptor binding protein (IGFBP) 4 (17661, US Biological, Swampscott, MA, USA; 1:3 dilution) and IGFBP5 (Ab4255, Abcam Cambridge, MA, USA; 1:300 dilution). After primary antibody incubation, sections were washed in Tris/NaCl buffer for 10 minutes. A Streptavidin-biotin multilink method (StrAviGen Multilink kit; Biogenex, San Ramon, CA, USA) was used for detection. The sections were incubated with secondary multilink antibody (1:20 dilution for 30 minutes) followed by a horseradish-peroxidase-labelled streptavidin complex (1:20 dilution for 30 minutes) at room temperature. Diaminobenzidine tetrachloride was applied for five minutes prior to washing in water for two minutes. Slides were then counterstained in hematoxylin, dehydrated and mounted. Expression was measured using a scoring system consisting of the product of the percentage of positive cells and their intensity of staining (0 to 3) producing a Histoscore ranging from 0 to 300. All tumor cells in the section were counted in the scoring system. Sections were scored by three independent readers and mean values obtained. Where initial scoring produced a value divergent by more than 10%, these sections were rescored until agreement was reached.

## Results

### Dynamic changes in gene expression produced by tamoxifen

**Figure 1:**
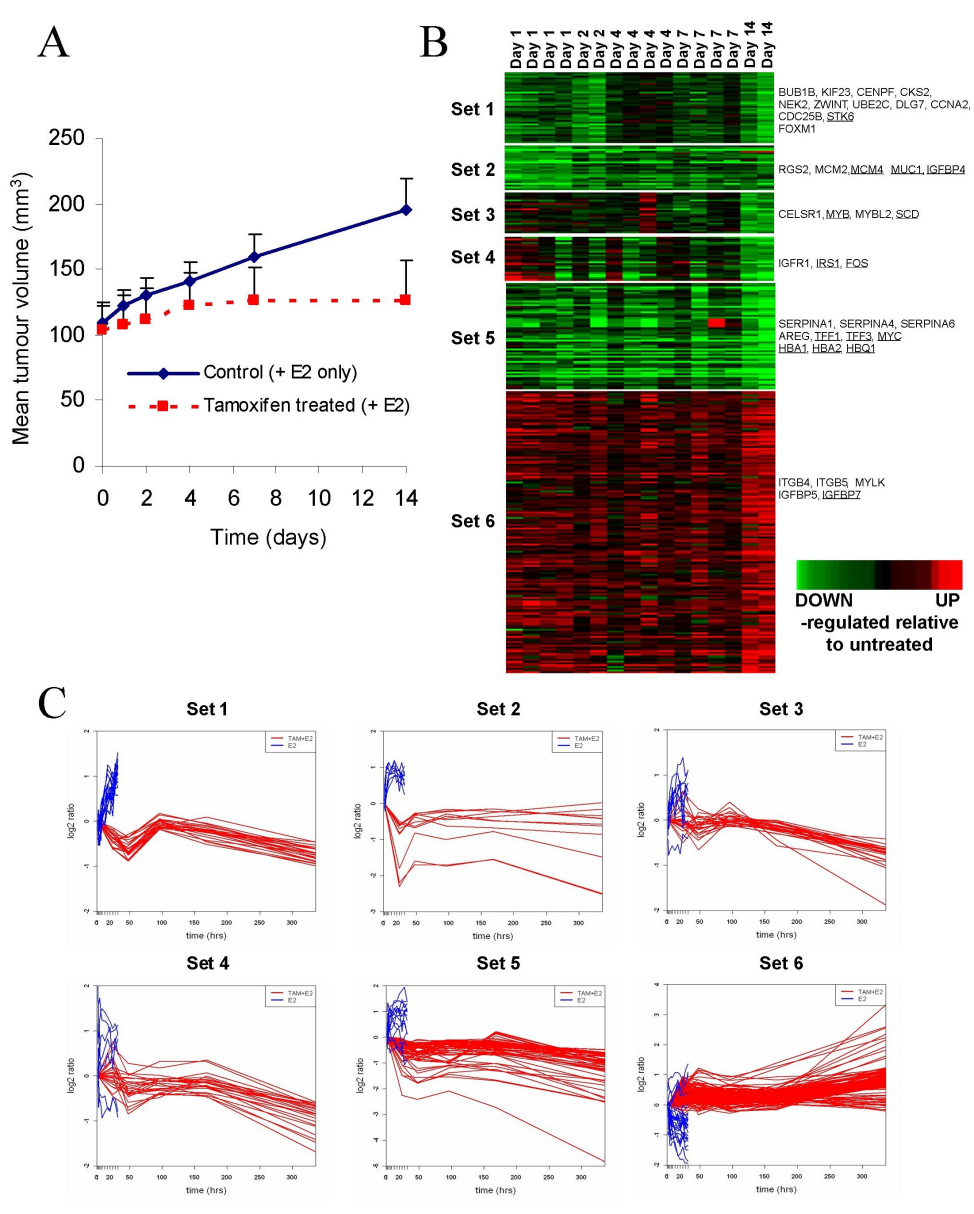
**Changes in gene expression over time in tamoxifen-treated xenografts**. **(a) **Comparison of the change in tumor volume over time in tamoxifen-treated and untreated (estrogen-supplemented) xenograft tumors. Values are the mean of four xenografts and error bars represent the standard error. **(b) **Heatmap illustrating genes with significantly increased (red) or decreased (green) expression in response to tamoxifen in the xenografts relative to no treatment. Underlined genes are those predicted to have estrogen-response elements (EREs) in their promoter regions. **(c) **Change in mean expression level (log2 fold change) of genes over time in xenografts treated with 17β-estradiol and tamoxifen (red). The changes shown in blue are those reported by Mutarelli and colleagues for 17β-estradiol alone [10].

The effect of tamoxifen on tumor volume growth and gene expression were studied on days 1, 2, 4, 7 and 14 after initiation of tamoxifen treatment and compared with tumors grown in the absence of tamoxifen. Tumor volumes were expressed relative to the initial tumor volume (Figure 1a). A reduction in tumor volume was clearly evident at day 7 and by day 14 the curves had significantly diverged. The graphs are significantly different at day 14 and are diverging by day 7 (*P *< 0.05; Student's t-test).

Across the five time points, 333 probes representing 253 genes showed evidence of at least a 1.5-fold change in level of expression (using a *P *≤ 0.05) [full list in the Additional File 1]. There was good agreement between the expression levels of xenograft replicates at most time points and the pattern of expression of these genes over the five time points was most consistently separated into six sets using hierarchical clustering (Figure 1b). These six sets of differentially expressed genes can be divided into three general groups: early/transient response (sets 1 and 2), variable response (sets 3 and 4) and continuous/late response (sets 5 and 6), relative to untreated samples. The early/transient-response genes were repressed relative to untreated samples, the variable-response genes were initially induced and then repressed and the continuous/late-response genes were both repressed (set 5) and induced (set 6). A large percentage of the genes in set 1 were very strongly associated with cell cycle regulation, among them AURKA, BUB1B, CCNA2, CDC25B, CDKN3, CENPF, CDC28, CKS2, DLG7, MKI67, NEK2, PRC1, STMN1, TACC3, UBE2C and ZWINT. BUB1, CKS2, PRC1, UBE2C and ZWINT have previously been shown to be estrogen-regulated in model systems [27]. Set 2 genes included mini-chromosome maintenance (MCM) 2 and MCM6, components of the replication fork [28], which may account for a primary response soon after treatment reducing DNA replication and regulation. Another member of set 2 was IGFBP4, which has been widely detected in breast tumors and cell lines, and previously correlated with ER expression [29].

Most of the variable-response genes in sets 3 and 4 responded rapidly to tamoxifen treatment, although they were both up- and down-regulated with some variation between replicates. The genes in set 3 were predominantly involved in cell proliferation, adhesion, and apoptosis including BTG2, MYB, MYBL2 and CELSR1, whereas genes in set 4, such as IRS and IGFR1 are involved in insulin receptor signalling. Set 5 and set 6 represent genes with the greatest down- and up-regulation at day 14, respectively (Figure 1b). Set 5 contained many classical ER-response genes including TFF1, TFF3 and MYC. Serpins A1, A4 and A6 were also strongly down-regulated. These genes play a key role in the control of tissue homeostasis and have previously been shown to be up-regulated in response to E_2 _in normal human breast tissue [30]. The cluster of up-regulated genes in set 6 was the largest cluster representing a wide variety of signalling pathways and processes. EREs were found in the promoter regions of a similar proportion (34 to 42%) of all six clusters of genes [genes shown in bold in the Additional File 1]. Studies by Carroll and colleagues have shown that ERs only sometimes regulate genes using EREs from proximal promoter regions and generally use distal enhancers and other binding sequences, such as Forkhead binding sites [31]. The observation of 34 to 42% of genes containing EREs in their promoter regions is consistent with these studies.

### Tamoxifen response compared with the response to estradiol over time

Many of the genes identified as changing in response to tamoxifen have also been identified in previous single time-point experiments, either in the opposite direction in response to 17β-E_2 _or in the same direction with tamoxifen in both *in vivo *and *in vitro *studies [8,30]. In order to establish whether the dynamic changes observed in this study reflected the reverse of the response to E_2 _over time, we compared our results with those from an *in vitro *time-course experiment, which also utilized the ZR-75-1 cell line [10]. Although that study had 12 time-points, with the final one being at 32 hours following addition of E_2_, the vast majority of genes showed the expected reciprocal changes in expression to those seen in the six clusters for the initial time points following treatment with tamoxifen in the present study (Figure 1c).

### Gene expression changes *in vitro*

**Figure 2:**
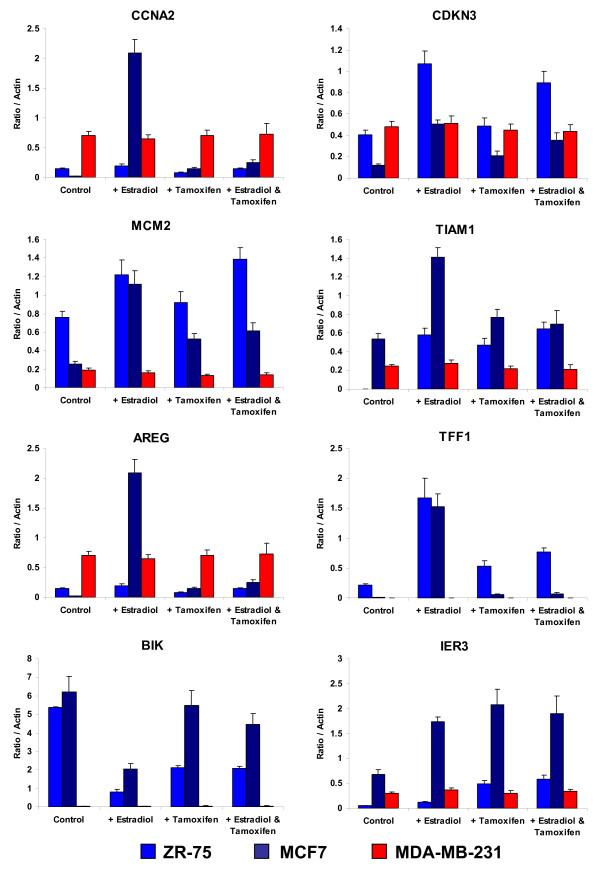
***In vitro *gene expression in two ER+ and one ER- cell line at 24 hours following treatment with tamoxifen**. Quantitative RT-PCR results for ZR75 (royal blue), MCF7 (dark blue) and MDA-MB-231 (red) with no treatment (control), addition of estradiol, tamoxifen or estradiol plus tamoxifen (changes at 6 hours and further genes shown in Additional File 2). ER, estrogen receptor.

To obtain further confirmation that the expression changes observed in response to tamoxifen were valid, 15 genes were selected for in vitro validation. These were analyzed in ZR-75-1 cells treated with either 0.1 nM E_2 _or 1 μM tamoxifen or both agents together to assess whether the genes were not only tamoxifen-regulated but also estrogen-regulated and whether tamoxifen was antagonising the estrogen-modulation or working via some other mechanism. A second ERα-responsive cell line, the MCF-7 line, was also used to assess whether the expression changes could be observed in an independent genotype. The ERα-negative cell line, the MDA-MB-231 line, was used to assess the specificity of these changes to involvement of ERα. Expression changes were measured at both 6 and 24 hours [Additional File 2]. The gene expression changes that were observed *in vivo *were also observed in these *in vitro *experiments and the changes seen in ZR-75-1 cells were mirrored in MCF-7 cells (Figure 2). Rather than reversing the expression change produced by E_2_, IER3 produced a greater change in the same direction. In contrast, there were no significant changes for any of these genes in the MDA-MB-231 cell line (Figure 2).

### Dynamic changes in protein expression within the ZR-75-1 xenograft model

**Figure 3:**
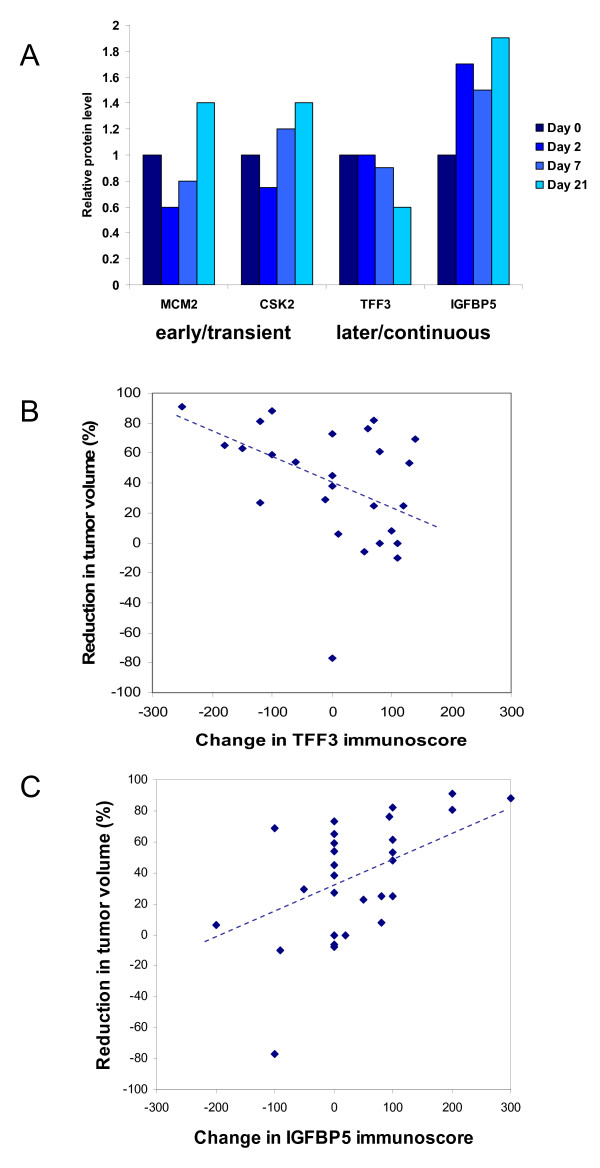
**Temporal protein expression of genes identified to respond to tamoxifen *in vivo***. MCM2, CKS2, IGFBP5 and TFF3 have similar expression at the protein level in response to tamoxifen in the ZR-75 xenograft by semi-quantitative immunohistochemistry. **(a) **They represent two pairs of examples of early/transiently and later/continuously responding proteins respectively. The correlation between the change in expression of proteins identified in the study and change in tumor volume in 28 patients treated with tamoxifen was calculated. Protein levels were scored by immunohistochemistry in tumor samples taken before and three months after treatment with tamoxifen. Changes in protein score are plotted relative to reduction in tumor volume for **(b) **IGFBP5 and **(c) **TFF3.

Four candidate genes were selected to evaluate whether expression changes at the protein level over time within this xenograft model were consistent with those seen at the gene expression level (Figure 3a). MCM2 and CKS2 were chosen as examples of early/transiently changing cell cycle-associated genes (sets 1 and 2). IGFBP5 and TFF3 were selected as examples of late/continuously up- and down-regulated genes, respectively (sets 5 and 6). Sections of the xenografts were assessed by semi-quantitative immunohistochemistry and histoscores related to the initial values. The protein expression of MCM2 and CKS2 had a similar profile to that seen at the gene expression level, although protein expression was higher at day 21 than at 0 and 14 days with gene expression. TFF3 expression decreased with time whereas IGFBP5 expression increased (Figure 3a).

### Protein expression changes in breast cancers treated with tamoxifen

To establish whether the change in expression of identified proteins correlated with changes seen *in vivo*, a series of primary breast cancers for which material was available pre- and post-tamoxifen treatment and in which the response at three months had been measured were analyzed by immunohistochemistry. Four proteins were selected (IGFBP5, TFF3, IGFBP4 and PDZK1) to represent the early/transient and later/continuous patterns of expression seen at the transcript. Breast cancer samples pre- and post-tamoxifen treatment were available for 28 patients and information on the percentage change in tumor volume was known. Change in histoscores in pre- and post-treatment paired samples were compared with the change in tumor volume (Figure 3b). The change in IGFBP5, TFF3 or both was significantly associated with change in tumor volume (*P *= 0.0135, 0.018 and 0.0002, respectively; Spearman rank test). This contrasted with data for IGFBP4 and PDZK1 where there was no significant association. PDZK was selected as a known estrogen-regulated gene that has been identified within a number of clinical data sets [32,33].

### Are the dynamically changing genes able to predict prognosis?

To evaluate whether the genes identified as dynamically changing in response to tamoxifen are associated with long-term follow up we downloaded four Affymetrix primary breast tumor datasets [11,34,35] from the NCBI GEO for patients that had all been treated with tamoxifen and for whom corresponding outcome data were available (Table 1). Affymetrix probesets representing the genes in the six gene sets with similar temporal profiles of expression were identified and clustered to separate tumors with high or low expression of each set of representative probesets [Additional File 3], as described previously [36]. Kaplan Meier plots were generated and log rank (Mantel-Cox) statistics calculated to see if the level of these sets of genes could discriminate between patients with good or poor outcomes. The set 1 cluster of genes was highly prognostic with all four datasets. Set 2, set 4 and all six gene sets combined also had some predictive power, although this was not consistent across the four datasets (Table 1). The genes driving this prognostic separation appear to be those involved with cell cycle and proliferation, patients with high levels of these genes at presentation are known to be at high risk of recurrence [13]. Additional File 3 illustrates the level of expression of the tamoxifen-response genes in primary tumors at presentation. Of the genes in set 1, one-third (11 out of 32) are represented in the 97 gene Genomic Grade Index that is undoubtedly associated with prognosis [35]; set 1 genes were also able to clearly distinguish between the histological grade of the tumors.

**Table 1:** Prognostic capacity of the sets of dynamically changing genes in patients treated with tamoxifen

Study/dataset	Tamoxifen-treated datasets				Untreated datasets	
	**Zhang and colleagues **[11]	**Loi and colleagues **[34]		**Sotiriou and colleagues **[35]	**Wang and colleagues **[37]	**Desmedt and colleagues **[38]
NCBI GEO dataset [20]	GSE12093	GSE6532		GSE2990	GSE2034	GSE7390

Affymetrix GeneChip	U133A	U133A	U133 plus2	U133A	U133A	U133A

No tumors (All ER+ and TAM-treated)	136	119	87	62	209	134

Tumor grade (1/2/3/NA)	8/43/30/55	1/94/4/20	17/37/16/17	32/0/27/3	NA	29/68/35/2

Age (median)	64*	65	63	66	52	47

Follow up (median)	7.1	5.2	11.4	4.9	7.2	10.4

Endpoint	DFS	RFS	RFS	RFS	RFS	DFS

All dynamic genes (sets 1 to 6)	***P ***= 0.7	***P ***= 0.2	***P *= 0.0006**	***P ***= 0.1	***P *= 0.005**	***P ***= 0.6

Set 1 (early/transient)	***P *= 0.00005**	***P *= 0.0002**	***P *= 0.0002**	***P *= 0.002**	***P *= 0.002**	***P *= 0.04**

Set 2 (early transient)	***P ***= 0.4	***P ***= 0.3	***P ***= 0.5	***P *= 0.03**	***P *= 0.03**	***P ***= 0.2

Set 3 (variable)	***P ***= 0.8	***P ***= 0.7	***P ***= 0.2	***P ***= 0.6	***P *= 0.0005**	***P ***= 0.1

Set 4 (variable)	***P ***= 0.5	***P ***= 0.2	***P *= 0.008**	***P ***= 0.6	***P ***= 0.7	***P ***= 0.3

Set 5 (continuous/late)	***P ***= 0.1	***P ***= 0.2	***P ***= 0.1	***P ***= 0.5	***P ***= 0.1	***P ***= 0.2

Kaplan-Meier (Mantel-Cox log rank) analysis of the endpoints; *Mean value. DFS, disease-free survival; ER, estrogen receptor; NA, not available; RFS, relapse-free survival; TAM, tamoxifen.

### Do the genes that are most changed at independent time points following treatment with tamoxifen predict prognosis?

**Figure 4:**
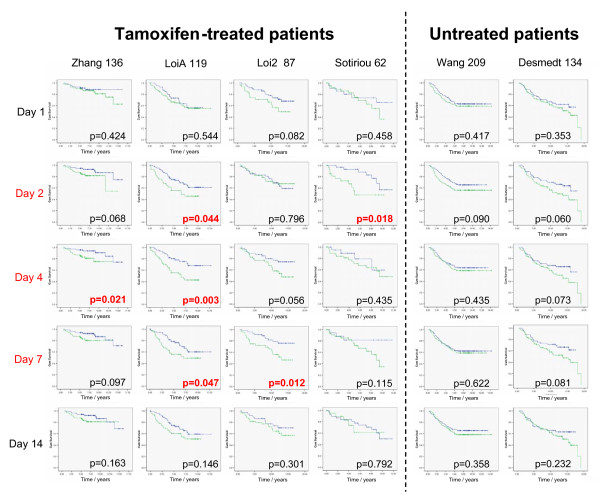
**Prediction of prognosis of tamoxifen-treated tumors based upon the 50 highest responding genes at each independent *in vivo *time point**. Kaplan-Meier analysis of four tamoxifen-treated and two untreated datasets [11,34,35,37,38], named by first author and the number of estrogen receptor (ER)-positive tumors with follow-up information (Table 1). Lists of genes are in Additional File 1. Green = primary tumors at presentation with expression profiles most like treated xenografts; Blue = primary tumors at presentation with expression profiles less like those of tamoxifen-treated xenografts.

Different numbers of probes were identified to be significantly differentially expressed at each time point. In order to compare the relative prognostic value of a profile of genes that are most changed at an individual time point following treatment, we identified lists of 50 probes with the greatest fold changes (up or down) at each time point among the list of 333 most changed probes as described above. Some probes were most changed at more than one time point [see Additional File 1]. Patients whose gene expression profile at presentation was more like that of xenograft tumors following treatment had a poorer prognosis [see Additional File 3]. Figure 4 demonstrates that profiles of the most differentially expressed genes at two, four and seven days following tamoxifen treatment were able to predict prognosis, while lists of genes most changed initially (day 1) or later (day 14) cannot. None of these most changed gene lists were significantly prognostic in two datasets [37,38] of ER-positive tumors that did not receive adjuvant therapy (Figure 4). Supervized principle components analysis [23] was also used to identify which genes within the profiles have the greatest prognostic power. The genes changed at each of the five independent time points were dominated by the late/continuously up- and down-regulated genes (sets 1 and 2), with early changes (sets 5 and 6) less represented and the transient changes hardly represented at all [see Additional File 1]. Known estrogen-response genes including IGFBP5, TFF3, TFF1, PDZK1 and SERPINA genes appear to dominate in prognostic performance. IGFBP5 expression is higher in patients with poor prognosis, as noted previously [29,39]; however, it is not significantly changed at day one, but is at subsequent time points, as seen at the protein level (Figure 3a). The heatmaps in Additional File 3 also indicate that IGFBP5 may be a good biomarker of outcome on tamoxifen.

## Discussion

This study is, to the best of our knowledge, the first to define tamoxifen-regulated gene expression profiles at multiple time points after long-term anti-estrogen treatment in an *in vivo *model of ER-positive breast cancer. The use of multiple time points over the 14-day period allowed analysis of the temporal patterns of gene expression profiles. Three basic patterns of change were observed; early/transient changes, continuous/late changes and more variable changes. The pattern of expression of representatives of these sets of genes was confirmed by quantitative RT-PCR and at the protein level by semi-quantitative immunohistochemistry. The changes observed in the expression of IGFBP5 and TFF3 correlated with reductions in tumor volume in primary tumors treated with tamoxifen. Two different approaches were used to evaluate whether those genes for which there was clear evidence of tamoxifen-induced changes in expression level would themselves be prognostic for patients treated with adjuvant tamoxifen. The early/transient pattern of gene expression associated with a reduction in cell cycle/proliferation genes and genes that were most differentially expressed on days two, four and seven were able to predict prognosis of primary breast tumors treated with tamoxifen. The IMPACT (Immediate Preoperative Arimidex Compared to Tamoxifen) trial demonstrated that Ki67 level two weeks after treatment was predictive of long-term outcome [40]. The timing of measurement of gene expression changes appears to be critical for certain groups of genes. A number of neoadjuvant 'window of opportunity studies' are underway to characterize changes in gene expression in response to treatment and establish whether clinical response after a couple of weeks or several months is predictive of long-term outcome. Studies such as that described here may provide insights as to when significant changes are detectable and which genes may represent good markers of response. It also highlights the risk that clinical snapshots of treatment could miss informative changes in expression.

There have been multiple short-term studies of *in vitro *profiling after estrogen treatment, predominantly in MCF-7 cells [41,42] and also in ZR-75-1 cells [4,10]. In a study using the T47D model of ER-positive breast cancer, Harvell and colleagues stated that E_2 _regulates different genes in human breast tumor xenografts compared with the identical cells in culture [9]. However, Creighton and colleagues found that genes regulated by estrogen in breast tumor cells *in vitro *are similarly regulated *in vi*vo in tumor xenografts and human tumors [8]. Disparities between approaches may be the result of differences in time points as well as the differences in microenvironment. Our *in vitro *study allowed exploration of whether tamoxifen's effects were antagonistic to estrogen or not. Fifteen genes were selected and all were modulated by estrogen *in vitro*. Of these genes, tamoxifen reversed the estrogen modulation in 14 cases but not for IER3. This gene was of particular interest in that while it was estrogen up-regulated, tamoxifen produced a greater up-regulation, it was also continuously up-regulated at all five time points and among the genes of set 6.

Our previous study [3] demonstrated early changes in apoptotic and mitotic indices (days 2 and 4) predated tumor volume changes, we speculate that the earlier/transient expression changes observed are more likely to be causative and primary events for tumor volume inhibition whereas later/continuous expression changes are possibly only consequential and secondary to the volume changes. These may represent changes in stromal elements and infiltrating cell populations. Ongoing studies are seeking to develop a putative model of how the early/transient changes interact with the later/continuous changes.

Many of the breast cancer gene expression signatures that have previously been developed highlight a number of genes associated with cell cycle and proliferation [35,36,43-46], which has been suggested to be largely a reflection of tumor grade. These genes appear to have most prognostic value for ER-positive breast tumors, generally differentiating between luminal A and luminal B subtypes, both prior to or following treatment with tamoxifen [13] or chemotherapy [47,48]. Our results are consistent with this idea and the suggestion that a lower response to E_2 _or growth factor signaling [49], also a feature of luminal B tumors, may also be prognostic. It is not clear from our results the extent to which prognosis or prediction of response to therapy is an intrinsic property of tumors.

Relatively high levels of the set 1 pattern of dynamically changing cell cycle/proliferation genes at presentation in primary tumors was associated with poor prognosis; however, it was relatively low levels of the classically up-regulated E2-response genes (down-regulated by tamoxifen), such as TFF1, TFF3, AREG and IGFBP4, at presentation that were among the genes most changed at day 4 and associated with poor prognosis. Conversely, a reduction in TFF3 (or an increase in IGFBP5) following tamoxifen treatment *in vivo *and the protein levels in primary tumors correlated with a reduction in tumor volume in the 28 patients treated with tamoxifen for three months. This apparent contradiction between the direction of change in genes upon treatment and their relative level in primary tumors as long-term predictors of outcome may be due to the complexity of estrogen signaling, the agonistic and antagonistic roles of estrogen and tamoxifen on the ER and/or a difference between short- and long-term effects on both tumors and normal tissues. We also recently demonstrated that proliferation genes were strongly down-regulated following treatment with the mTOR inhibitor, everolimus, despite these often being considered markers of prognosis [50].

The aim of this study was to assess the dynamic response to tamoxifen, not to find the definitive tamoxifen-response signature or biomarker. A better test of the tamoxifen-response genes in primary tumors would be a dataset from a neoadjuvant 'window study' [15] of gene expression before and after tamoxifen with both clinical or pathological endpoints and long-term follow-up. It would be interesting to measure gene expression at multiple time points in a number of different cell line xenograft models or primary tumors in order to fully investigate patient-patient variation in temporal response to tamoxifen. This approach would also benefit from single-color microarrays in order to evaluate the relative merits of pre- and post-treatment samples avoiding the limitation of using comparative two-colour cDNA arrays, as in this study. We did examine gene expression of the different response patterns (sets 1 to 6) and individual time points in matched before and after breast biopsies from patients treated with 14 days of neoadjuvant letrozole [51] and found largely consistent changes for most genes with those of the *in vivo *study in the majority of cases (data not shown). Further work is required to fully assess how the response to different hormonal therapies and short-term molecular changes correlate with long-term outcome. We have previously demonstrated that estrogen-regulated gene expression predicts response to endocrine therapy in patients with ovarian cancer [27], and in this study we demonstrate for the first time that tamoxifen-response genes identified from a xenograft breast cancer model with different profiles of expression can predict prognosis in primary tumors treated with tamoxifen.

The genes highlighted in this study are now being explored in clinical material collected by biopsy from patients pre- and post-treatment with tamoxifen and who are known to have either responded to or progressed on treatment. This will help determine which of the genes identified in this study have the potential to be predictive markers of response. This study also suggests that future studies searching for genes predictive of outcome on therapy could perhaps be informed by studies that identify which genes demonstrate early dynamic response to therapy, rather than those with sustained changes. This is reminiscent of data from early positron emission tomography (PET) scans that suggest the patients with the best outcome on therapy are those with pronounced early reduction in PET signal [52,53].

## Conclusions

Both early/transient/proliferation-response genes and continuous/late/estrogen-response genes are able to predict prognosis of primary breast tumors in a dynamic manner. Temporal expression of therapy-response genes is clearly an important factor in the response to endocrine therapy in breast tumors which has significant implications for the timing of biopsies in neoadjuvant biomarker studies.

## Abbreviations

CKS: CDC28 protein kinase regulatory subunit; DAVID: Database for Annotation, Visualization and Integrated Discovery; DMEM: Dulbecco's modified Eagle's medium; E_2_, estradiol; ER: estrogen receptor alpha; ERE: estrogen-response elements; FCS: fetal calf serum; IGFBP: insulin growth factor receptor binding protein; MCM: mini-chromosome maintenance; NCBI GEO: National Centre for Biotechnology Information Gene Expression Omnibus; PET: positron emission tomography; RT-PCR: reverse-transcription polymerase chain reaction; TFF: trefoil factor.

## Competing interests

The authors declare that they have no competing interests.

## Authors' contributions

SPL, DAC and DH conceived and directed the study. KT undertook the microarray and RT-PCR studies. GW, BK and SPL performed the immunohistochemistry on the clinical breast cancer cases. DAC obtained tissue blocks. AHS and LL analyzed the microarray data. MM carried out the xenograft work. DF undertook the immunohistochemistry on the xenograft material. JMD collected the clinical material. KT, AHS and SL drafted the manuscript. All authors read and approved the final manuscript.

## Supplementary Material

Additional file 1 **Lists of differentially expressed genes**. Microsoft Excel Workbook containing probe and gene names, plus Ensembl identifiers and mean fold changes for the 333 significantly differentially expressed probes. Also provided are lists of the 50 most changed genes at the five timepoints following treatment with tamoxifen. Click here for file

Additional file 2 **Quantitative PCR results**. Gene expression *in vitro *measured by quantitative RT-PCR for ZR75 (royal blue), MCF7 (dark blue) and MDA-MB-231 (red) before (0), 6 and 24 hours following no treatment (C), addition of estradiol (E), tamoxifen (T) and estradiol plus tamoxifen (ET). Click here for file

Additional file 3 **Examples of heatmap clustering**. Heatmaps showing the level of expression of the **(a) **set 1 and **(b) **day 4 tamoxifen-response genes in primary tumors at presentation. Patients whose expression of set 1 genes correlate with post-treatment xenograft samples have a good prognosis (blue). However, patients whose expression of genes at presentation is more like those that were differentially expressed at day 4 following tamoxifen treatment tend to have a poor prognosis (green). See Table 1 and Figure 4 for survival analysis results. Click here for file
