# Supplementary figures and images for: Dynamic changes in gene expression in vivo predict prognosis of tamoxifen-treated patients with breast cancer
Source: Breast Cancer Res. 2010 Jun 22;12(3):R39. doi: 10.1186/bcr2593 (PMC2917034; doi:10.1186/bcr2593)

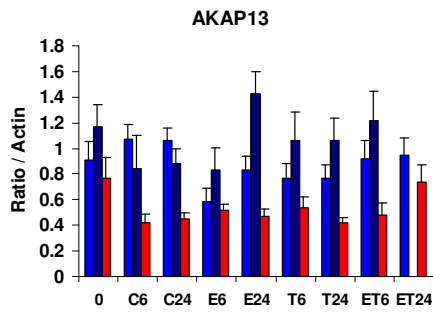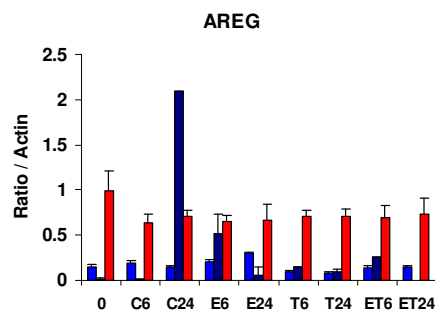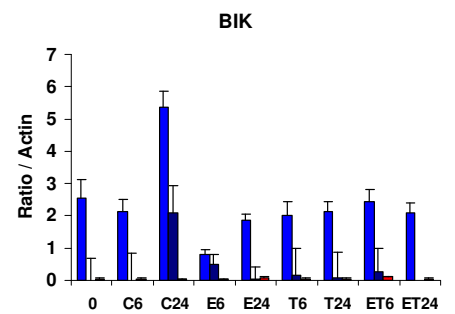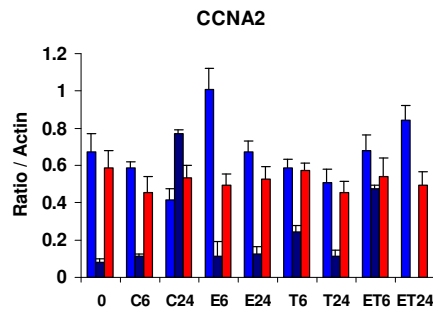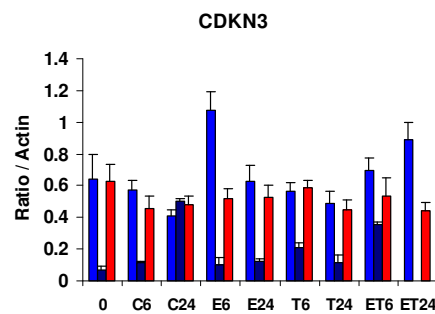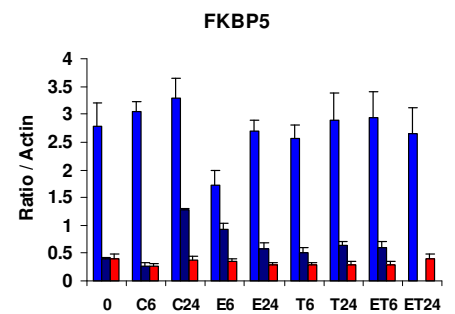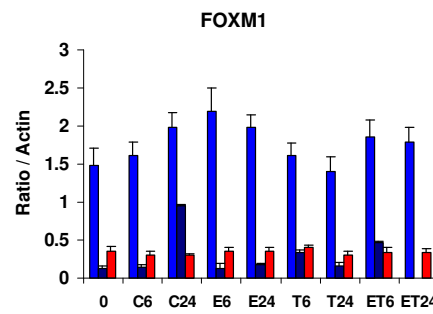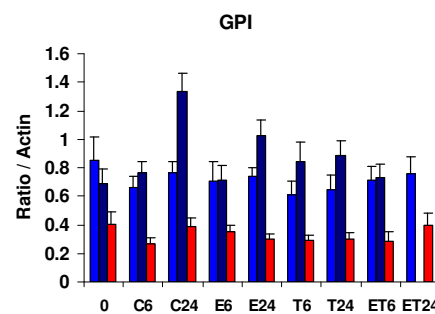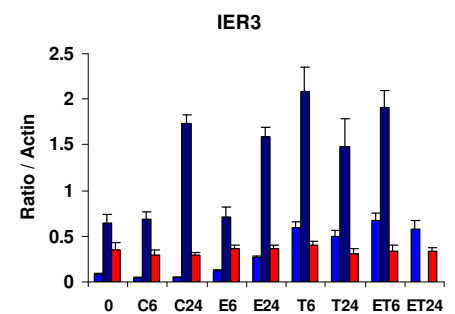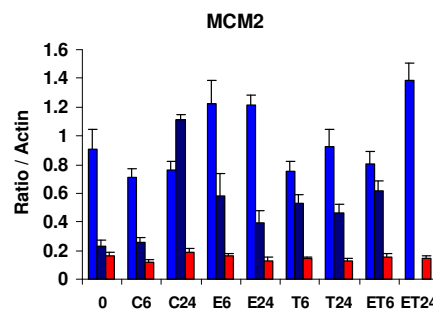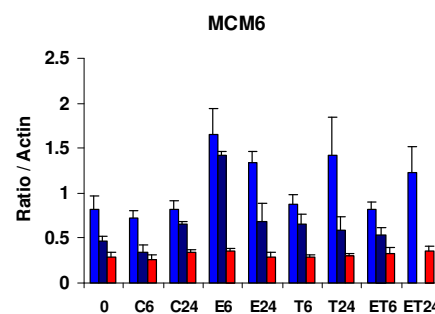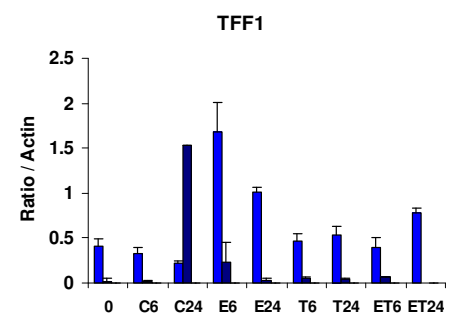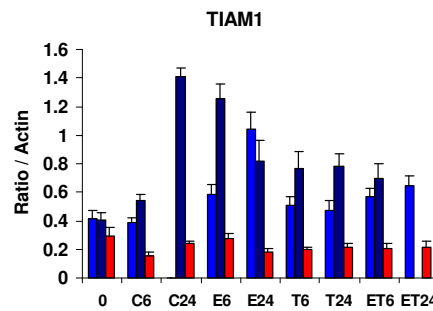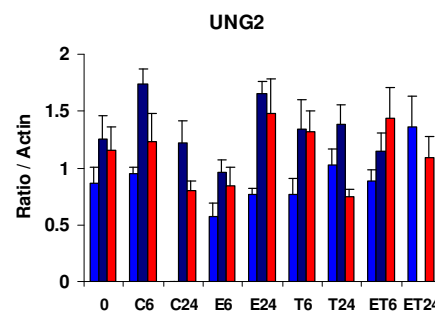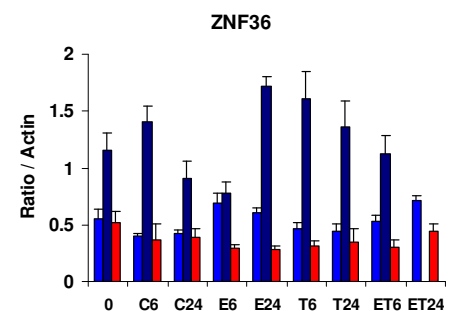

Supplement: Additional file 2 — Quantitative PCR results. Gene expression in vitro measured by quantitative RT-PCR for ZR75 (royal blue), MCF7 (dark blue) and MDA-MB-231 (red) before (0), 6 and 24 hours following no treatment (C), addition of estradiol (E), tamoxifen (T) and estradiol plus tamoxifen (ET). [file bcr2593-S2.PDF]
