# Supplementary material for: Dynamic changes in gene expression in vivo predict prognosis of tamoxifen-treated patients with breast cancer
Source: Breast Cancer Res. 2010 Jun 22;12(3):R39. doi: 10.1186/bcr2593 (PMC2917034; doi:10.1186/bcr2593)
Supplement: Additional file 3 — Examples of heatmap clustering. Heatmaps showing the level of expression of the (a) set 1 and (b) day 4 tamoxifen-response genes in primary tumors at presentation. Patients whose expression of set 1 genes correlate with post-treatment xenograft samples have a good prognosis (blue). However, patients whose expression of genes at presentation is more like those that were differentially expressed at day 4 following tamoxifen treatment tend to have a poor prognosis (green). See Table 1 and Figure 4 for survival analysis results. [file bcr2593-S3.PDF]

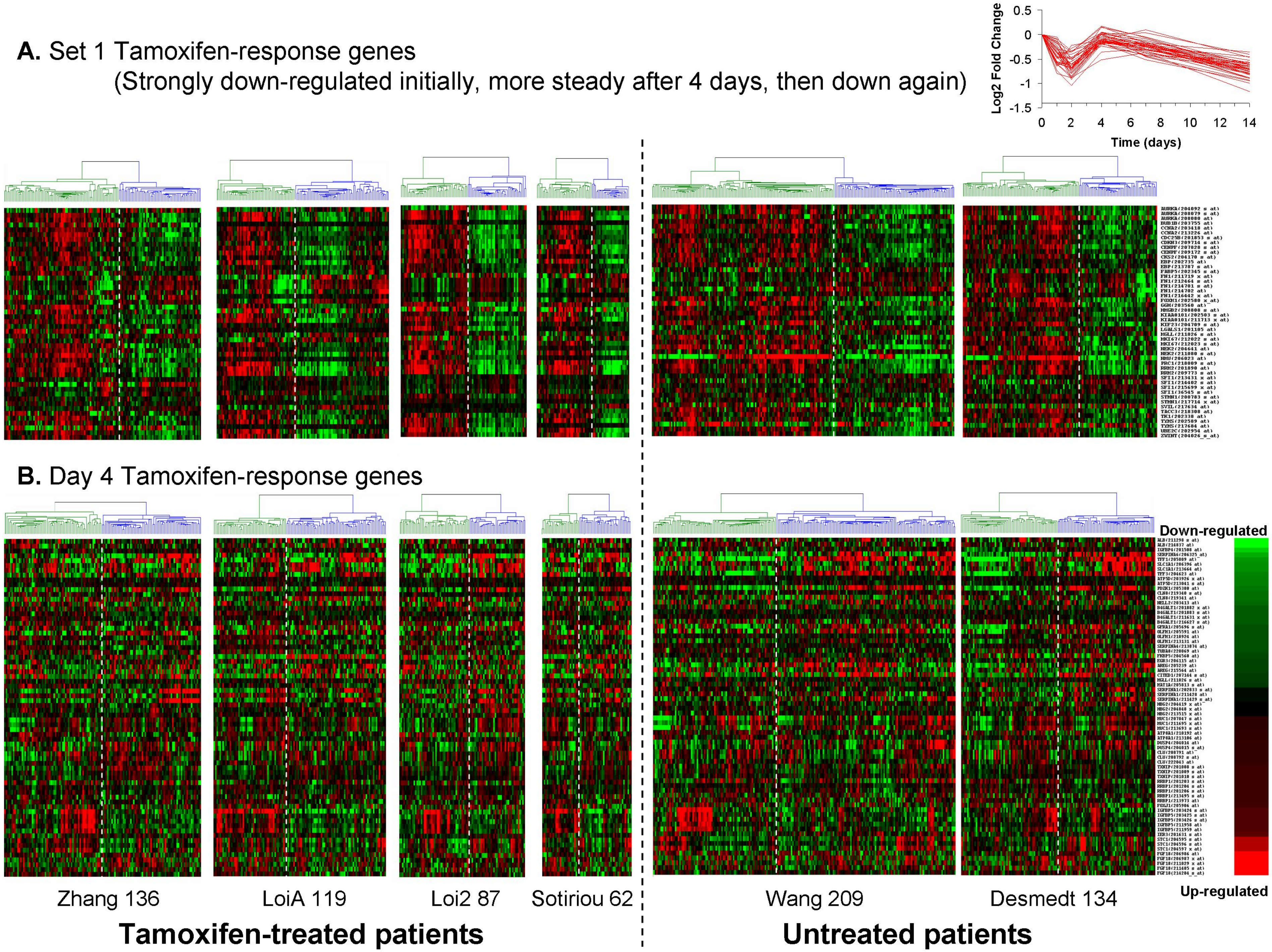
A. Set 1 Tamoxifen-response genes
(Strongly down-regulated initially, more steady after 4 days, then down again)
Log2 Fold Change
0.5
0
-0.5
-1
-1.5
0 2 4 6 8 10 12 14
Time (days)
B. Day 4 Tamoxifen-response genes
Down-regulated
Up-regulated
Zhang 136
LoiA 119
Loi2 87
Sotiriou 62
Wang 209
Desmedt 134
Tamoxifen-treated patients
Untreated patients
